# Hereditary Hemorrhagic Telangiectasia—A Case Series Experience from a Liver Transplant Center in Romania

**DOI:** 10.3390/diagnostics12122959

**Published:** 2022-11-26

**Authors:** Christopher Pavel, Teodor Cabel, Dragoș Dinuță, Alexandru Zaharia, Simona Olimpia Dima, Vasile Sandru, Mugur Cristian Grasu, Mariana Mihaila

**Affiliations:** 1 Department of Gastroenterology, Emergency Clinical Hospital Bucharest, 014461 Bucharest, Romania; 2 Department of Internal Medicine, Fundeni Clinical Institute, 022328 Bucharest, Romania; 3 Department of General Surgery and Liver Transplantation, Fundeni Clinical Institute, 022328 Bucharest, Romania; 4 Department of Interventional Radiology and Medical Imaging, Fundeni Clinical Institute, 022328 Bucharest, Romania; 5 Department of Radiology, Carol Davila University of Medicine and Pharmacy, 050474 Bucharest, Romania

**Keywords:** hereditary hemorrhagic telangiectasia—HHT, arteriovenous malformations—AVM, epistaxis, secondary hepatic vein dilatation

## Abstract

Hereditary hemorrhagic telangiectasia (HHT) has significant morbidity due to multiorgan involvement and an unpredictable disease course. We analyzed the data of 14 patients diagnosed with HHT. The case series comprised 14 patients with a median age at presentation of 48 years old (41–74 years). In twelve patients (85.7%), the diagnosis was confirmed by using the Curacao Criteria. The most common reason for admission was epistaxis, with 9 patients (57%) presenting with nosebleed refractory to prolonged self-tamponade. The biochemical abnormalities identified were elevations in AP and gamma-GT; liver synthetic function was generally normal, even though 21% of patients had clinical or imaging findings for cirrhosis. Nosebleeds were the main reason for admission and significantly impacted quality of life through anemia and frequent hospital admissions. However, the visceral manifestations seemed to be more serious. The hepatic arteriovenous malformations (AVMs) appeared to remain asymptomatic or led to minimal changes for the majority of patients; some cases were associated with liver and biliary tract ischemia, necrosis leading to acute liver failure and even death. Hepatic AVMs can also lead to high-output heart failure due to arterio-venous shunting. The most frequent AVM was hepatic artery to hepatic vein, with secondary hepatic vein dilation and hemodynamic consequences.

## 1. Introduction

Hereditary hemorrhagic telangiectasia (HHT), more frequently known in clinical practice as Osler–Weber–Rendu syndrome, is a rare autosomal dominant disorder with variable penetrance, characterized by telangiectasia or arteriovenous malformations (AVM) which affect multiple organs and systems (primarily involving the skin, mucosa, liver, lungs, and brain). The estimated worldwide prevalence ranges from 1 per 5000 to 1 per 10,000, but it is most probably underrated [1]. 

The clinical manifestations of HHT are varied and most patients experience manifestations of epistaxis, skin and mucosal telangiectasis, or iron deficiency anemia secondary to gastrointestinal bleeding. 

The percentage of patients with liver AVM in HHT was between 8–31% according to older studies [2]. However, current data suggest that the proportion of liver involvement in patients affected by HHT disease can exceed 70% [3]. The significant percentage (30–50%) of patients with vascular involvement of the liver who remain asymptomatic throughout the course of the disease may have an impact on the prevalence rate [2,4]. The prevalence of liver involvement may rise up to 79% when a CT scan is used to screen a large cohorts of patients [4,5].

Published data have shown that the incidence rates of death and complications are 1.1 and 3.6 per 100 persons per year [1,6]. HHT can involve specific and nonspecific liver lesions in forms ranging from small telangiectasia to large arteriovenous malformations. The hallmark lesion is represented by telangiectasia, a direct connection between the arteriole and venule which bypasses the capillary bed [7]. The different severities of liver vascular malformation explain the high variability of clinical symptoms [6].

Following genetic research, several mutations have been identified in HHT involving the endoglin (*ENG*) and activin receptor-like-kinase (*ACVRL1*) genes which encode receptors of the transforming growth factor-beta family (endoglin and activin receptor-like kinase-1, respectively). *ENG* mutations are more frequently observed in pulmonary and cerebral AVMs (type I HHT), while *ACVRL1* mutations are more prevalent in hepatic AVMs (type II HHT) [8]. Hepatic malformations are more frequent in HHT type 2 [1].

Considering the significant morbidity of the disease due to multiorgan involvement and the unpredictable disease course, we decided to analyze data of 14 patients diagnosed with HHT in the Internal Medicine Department of the Fundeni Clinical Institute

## 2. Materials and Methods

This case series covered the period between January 2012 and June 2022.

We collected data from 14 patients in the aforementioned period using Excel Microsoft v. 2019 for the descriptive analysis.

Every patient had a thorough biochemical profile, including CBC, renal and liver function tests, alkaline phosphatase, gamma-GT, coagulation tests, lipid profile, C-reactive protein, and serum electrolytes. Radiologic imaging, including ultrasound, computed tomography (CT), and/or magnetic resonance imaging (MRI), was used to evaluate the liver in HHT patients.

The diagnosis of HHT was performed in accordance with the International Guidelines for HHT [9], based either on a consensus clinical diagnostic in 12/14 patients (Curaçao criteria [10,11]) or by the identification of the causative gene mutation (2/14 patients). Regarding the clinical diagnosis, HHT was confirmed when at least three criteria were present, was possible or suspected when 2 criteria were present, and was unlikely when only 1 criterion was present in an adult patient. The Curaçao criteria are listed below.

1. Epistaxis (recurrent, spontaneous bleeding episodes with no prior traumatic involvement, night-time nosebleeds).
2. Telangiectasis—unevenly distributed on lips, nose, fingers, oral cavity.
3. Visceral involvement—AVM in the lungs, brain, liver, stomach, spinal cord.
4. A family history of HHT—first-degree relative with a prior diagnosis of HHT.

The patients with a long history of frequent recurrent nosebleeds were surveyed using the Epistaxis Severity Score (EES). The overall score ranges from 0 to 10 and the severity is categorized as:

- None: score 0 or 1;
- Mild: score 1 to 4;
- Moderate: score 4 to 7;
- Severe: score 7 to 10 [12].

## 3. Results

### 3.1. Case Series Population

The case series group comprised 14 patients (12 women and 2 men) with a median age at presentation of 48 years old, [IQR 41–74]. In twelve patients (85.7%), the HHT diagnosis was confirmed by using the Curacao Criteria (at least 3 out of 4 consensus criteria being fulfilled). All these patients (12/12) had at least one first-degree family member previously diagnosed with HHT; furthermore, one-quarter of patients (4/12) had two or even more family members known to have HHT. In two patients with equivocal familial or clinical history, genetic testing was performed, revealing mutations in the *ACVRL1* gene.

### 3.2. Clinical Presentation and Lab Testing

The most common reason for admission was HHT-associated epistaxis, with nine patients (57%) presenting with nosebleed refractory to prolonged self-tamponade management—Table 1. 

The mean EES was 4.1 (range 1.9 to 6.6), which can be ascribed as moderate severity. In 7/9 patients with epistaxis, the application of topical vasoconstrictor agents or nasal packing was performed; one patient required endovascular embolization procedures and one patient underwent surgical nasal closure. The mean hemoglobin value in this subgroup was 9.6 g/dL (range 5.7 g/dl–11.6 g/dl) and two patients required single-unit blood transfusions. 

Two patients (14%) presented with low iron deficiency anemia and intermittent melena. An upper digestive endoscopy revealed multiple gastric and duodenal telangiectasias, eventually treated with argon plasma coagulation (APC) in two or three separate sessions due to the complexity of the lesions. Only one patient required a blood transfusion (up to five units) due to recurrent bleeding after the initial endoscopic hemostasis attempt. 

The other two female patients presented with intense right upper quadrant pain. In the first case, an abdominal CT scan revealed bilobate hepatic perfusion abnormalities due to diffuse AVMs with peribiliary ischemia and right lobe 6/5 cm biloma, most probably developed as a consequence of bile duct necrosis. In the second case, similar findings were noted, with even more extensive ischemic liver lesions, but without any hepatic collections. 

Exertional dyspnea was another admission reason for one patient (7%), and further investigations indicated high-output heart failure (a resting cardiac output greater than 8 L/min, ejection fraction >55%) due to multiple liver arteriovascular malformations (mainly arterioportal shunts). 

The main laboratory findings are summarized in Table 2.

The most common biochemical abnormalities were elevations in alkaline phosphatase and gamma-GT; liver synthetic function (albumin, protein synthetic, and prothrombin time) was generally normal, even though 3/14 (21%) patients had clinical and imaging findings compatible with the diagnosis of cirrhosis. In our report, we encountered two cases of acute liver failure with INR and bilirubin elevations (aside from clinical manifestations of hepatic encephalopathy) due to spontaneous biliary/hepatic necrosis. One death occurred (F, 47 years old) due to multiple-organ failure after a prolonged hospitalization in the ICU. Nevertheless, one patient (F, 42 years old) underwent liver transplantation with an excellent outcome during a follow up.

After a thorough clinical and biological examination, an abdominal CT and/or MRI scan was performed on 10/14 patients, a chest CT scan was performed on 8/14 patients, and a cerebral CT was performed on 6/14 patients, as shown in Table 3.

As summarized in the table above, the liver was the most common site of AVM. Considerably, no patient had only one vascular shunt, but multiple vascular malformations; in the majority of patients (10/14, 71%), the most common AVM was hepatic artery to hepatic vein shunting, with secondary hepatic vein dilation, which explains the hemodynamic consequences and the secondary high output heart failure. Most of the patients (10/14, 71%) presented hepatomegaly, and four of these patients were also associated with splenomegaly. One patient with multiple AVM and liver perfusion abnormalities was also associated with several hepatic round-shaped collections surrounding the biliary tree, resembling biloma. 

Portal hypertension manifestations were observed in 4/14 (28%) patients, with typical imaging findings (portal vein enlargement, splenomegaly, collateral vessels, mild ascites) but without esophageal/gastric varices; only two patients had thrombocytopenia. Biliary abnormalities were frequently noted, and 7/14 patients (50%) had elevated alkaline phosphatase and/or gamma GT; of these, 4 patients presented intrahepatic biliary dilatation on abdominal imaging. Multiple focal nodular hyperplasia lesions (FNH) were described in our review in a 68-year-old female patient.

**Figure 1:**
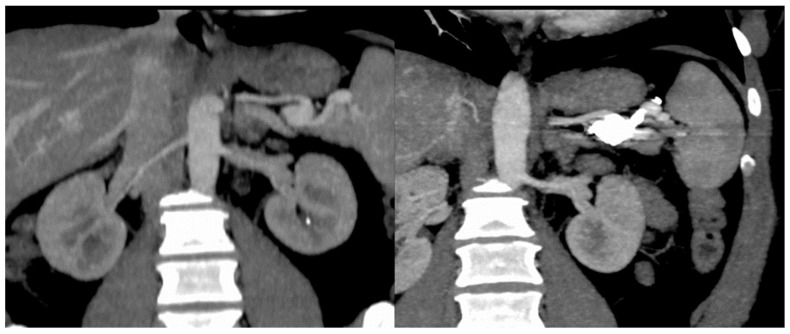
Embolization of splenic artery aneurysm in HHT patient.

In our case series, we detected two splenic artery aneurysms in the same patient, and one of them was considered to have a high potential for bleeding. Therefore, a radiological embolization was performed, with a favorable outcome. The procedural radiological imaging is listed in Figure 1.

We report that out of the 14 patients evaluated in this case series, 2 (14%) had cerebral imaging performed, none of which presented any vascular malformations, ischemic changes or signs of infection. Out of the eight patients investigated with a CT chest scan, two had multiple pulmonary AVMs with variable distribution while the other three patients had only isolated AVMs.

## 4. Discussions

The most common reason for admission was HHT-associated recurrent epistaxis and refractory to prolonged self-tamponade management. Only two patients required interventional treatments (endovascular embolization and surgical nasal closure), while the remaining patients were successfully managed by vasoconstrictors agents or nasal packing. Other therapies would have been questionable, as topical bevacizumab, a monoclonal antivascular endothelial growth factor antibody, had failed to be more effective in controlling epistaxis than classical alternatives in three randomized controlled trials [13]. 

Other patients presented with iron-deficiency anemia and melena due to upper gastrointestinal bleeding from multiple telangiectasias that required endoscopic hemostatic therapy with plasma argon (APC). Only one patient required a blood transfusion due to recurrent bleeding after initial endoscopic therapy. There are multiple prospective studies that have highlighted the importance of small bowel endoscopy examination; Proctor et al. performed an enteroscopy on 27 HHT patients and concluded that the presence and number of gastric/duodenal telangiectasia correlates with the presence and number of jejunal lesions [14]. In our case series, a balloon-assisted enteroscopy was performed on one patient, with one nonbleeding telangiectatic lesion identified in the jejunum. The HHT guidelines recommend an annual measurement of Hb and serum iron levels beginning at 35 years old and suggest that an endoscopic evaluation should be performed only in the case of anemia that is disproportionate to the amount of epistaxis [15]. The effectiveness of intravenous bevacizumab in reducing GI bleeding and transfusion needs is still controversial and reliable reports or RCT are awaited [16].

Exertional dyspnea was the main admission reason for one patient due to high output heart failure (HOHF). However, HHT-related HOHF was eventually reported in 8/14 patients (57%) after further evaluation (at our institution, echocardiography is part of the standard protocol for the evaluation of HHT patients). This is significantly higher than the 1.4 per 100 persons per years incidence of HOHF found by Buscarini et al in a prospective study of 502 HHT patients [6]. The pathophysiologic mechanism implies the shunting of oxygenated blood from the hepatic artery to the hepatic vein, bypassing the liver and decreasing effective perfusion. A liver transplant is the only definite treatment that reverses this condition [17]. In the past, a hepatic artery embolization was occasionally performed, but due to an unacceptably high rate of complications (liver necrosis, cholangitis), it has been abandoned [18]. In an international survey of 31 international HHT Centers, IV bevacizumab was reported to be effective in 55% of patients with significant improvement in cardiac index and HOHF symptoms, with uncommon adverse events (<10%); however, half of the centers reported difficulty with the insurance approval process [16]. 

We encountered three emergency admissions. Two cases were attributable to acute liver failure due to spontaneous biliary and hepatic necrosis. One patient was also associated with several hepatic round-shaped collections surrounding the biliary tree, resembling biloma; she was enlisted for liver transplant, but she died in the ICU several days after admission with rapidly developing multiple organ dysfunction syndrome (MODS). The proposed mechanism of biloma formation is biliary ischemia secondary to intrahepatic vascular shunting and hypoxia [19], since the main blood supply derives from the peribiliary plexus from the hepatic artery. The second case had also been enlisted for liver transplant and successfully underwent the procedure (brain-dead liver donor) with excellent outcomes in terms of evolution. 

Regarding arterial malformations, there are other brief reports which suggest the increased risk of developing arterial aneurysms, mainly splenic artery aneurysms (SAA) in patients with HHT [20]. Sellier et al found a 4.57 higher rate of SAA in their prospective study of 186 HHT patients who were screened with a multidetector CT [21]. In our case series we also encountered one patient with a splenic artery aneurysm that was successfully treated with embolization (Figure 1).

There are several case reports in the literature with successful liver transplantation in HHT patients; the main indications for transplant were postembolic complications, heart failure, and spontaneous biliary/hepatic necrosis [22]. According to Secondary International HHT Guidelines [9], the suggested MELD exception score for HHT includes a score of 40 for patients with acute biliary necrosis and 22 points for patients with high-output heart failure. The European Liver Transplant registry reported a survival of 80% in a median follow up of 58 months in 40 HHT patients; therefore, a liver transplant may be considered a reasonable option in carefully selected patients, as it was also proved in our case series.

Large pulmonary arteriovenous malformations may be present in 5–15% of patients with HHT. In such patients, massive hemoptysis is a potentially life-threatening condition. An angioembolization of these large pulmonary shunts by interventional radiologists can reduce the risk of massive hemoptysis during or after liver transplantation [23]. 

The goals of PAVM management are to minimize embolic complications, prevent and treat hemorrhage from PAVM rupture, and improve functional capacity by reducing shunt-related hypoxemia [24].

## 5. Conclusions

Osler–Weber–Rendu disease is a serious condition with a strong genetic component, affecting multiple organ systems, diminishing quality of life, and in some cases leading to fatality. A discussion with an integrated team of experts in HHT centers and a multidisciplinary approach should be considered for the invasive treatment of these patients.

## Figures and Tables

**Table 1:** Clinical manifestation of the 14 patients.

Patient No	Age	Sex	Curaçao Criteria	Molecular Diagnosis	Bleeding	Hepatic MANIFESTATIONS	Cardiac MANIFESTATIONS	Cerebral CT	Chest CT	Abdominal CT
1	47	F	+	−	+	+	+	+	n/a	+
2	68	F	+	−	+	+	+	n/a	n/a	+
3	54	M	+	−	+	−	+	+	n/a	n/a
4	41	F	−	+	+	−	−	n/a	+	+
5	43	M	+	−	+	+	−	n/a	+	+
6	74	F	+	−	−	+	+	+	n/a	+
7	51	F	+	−	+	−	+	n/a	n/a	n/a
8	42	F	+	−	−	−	+	+	+	n/a
9	45	F	+	−	+	+	+	+	+	+
10	49	F	+	−	+	−	−	n/a	n/a	n/a
11	52	F	+	−	+	+	−	n/a	+	+
12	64	F	+	−	+	−	+	n/a	+	+
13	51	F	+	−	−	+	+	+	+	+
14	40	F	−	+	+	−	−	n/a	+	+

+, positive/present; −, negative/not present; n/a, nonassessed; CT—computed tomography.

**Table 2:** The main laboratory findings for each patient with Osler–Weber–Rendu disease.

Patient No.	Hb (g/dL)	Platelets (/uL × 1000)	Feritin (ng/mL)	INR	GGT (U/L)	ALP (U/L)	AST (IU/L)	ALT (IU/L)	Cholesterol (IU/L)	Creatinine (mg/dL)
1	9	307	-	1.92	304	259	226	97	187	0.99
2	10	211	-	1.29	156	138	25	15	165	
3	7.5	235	20.2	1.13	40	76	22	22	198	1.15
4	7.1	338	3.6	1.07	20	64	24	28	187	0.86
5	15.6	303	43	0.96	16	68	20	21	198	0.97
6	12.3	31	85.6	1.43	450	160	77	29	157	1.37
7	11.6	300	14.3	1.12	20	103	27	25	189	1.01
8	11.1	432	27.7	1.06	122	219	19	16	176	0.74
9	10.7	355	6.7	1.08	39	85	25	30	177	0.63
10	12.4	182	8.5	0.96	5	46	19	14	191	0.84
11	6.8	222	6	1.2	208	231	25	22	185	0.8
12	5.7	126	45.9	1.75	75	70	22	27	168	0.93
13	9.7	255	21	0.91	39	97	29	40	230	0.79
14	11.4	381	20.2	0.97	3	70	24	16	245	0.78
Mean	10.35	277.5	20.2	1.1	39.5	91.1	24.5	23.5	187.0	0.9
Range	7.1–15.6	31.000–432.000	3.6–85.6	0.91–1.75	3–450	46–259	19–226	14–97	157–245	0.63–1.37

**Table 3:** The abdominal involvement for each patient with Osler–Weber–Rendu disease.

Patient No.	1	2	3	4	5	6	7	8	9	10	11	12	13	14	Total
Hepatomegaly	+	+	+	+	+	+	+	+	+	+	+	+	−	+	13
Splenomegaly	−	−	−	−	−	+	−	−	+	−	+	−	−	−	3
Cirrhotic liver	−	+	+	−	−	−	−	−	−	+	+	+	−	−	5
HA diameter (mm)	+	14	13	−	−	12	11	11	10	13	14	+	14	10	Mean −12.2
Dilated/tortuous intrahepatic artery branches	+	+	+	−	−	−	−	−	−	+	+	+	−	−	6
HA to HV shunting	+	+	+	+	+	+	−	+	+	−	+	+	+	−	11
HA to PV shunting	+	+	+	−	−	−	−	−	−	−	+	+	−	−	5
PV to PV shunting	−	+	+	−	−	−	−	−	−	−	−	−	−	−	2
Intrahepatic telangiectasis	+	+	+	+	+	+	−	+	+	+	+	+	+	−	12
Splenic artery thrombosis	+	−	+	−	−	−	−	−	+	−	−	−	−	−	3
Heterogenous enhancement on arterial phase	+	+	+	+	+	−	−	+	+	+	+	+	−	−	10
PV enlargement	−	+	+	−	−	+	−	−	+	−	+	+	−	−	6
HV enlargement	+	+	+	+	−	−	−	+	+	+	+	+	−	−	10
Collateral circulation	+	+	−	−	−	−	−	+	+	−	+	−	−	−	5
IHBD dilatations	−	+	+	−	−	+	+	−	−	+	−	−	−	−	5
Biliary cyst	−	−	−	−	−	−	−	+	−	−	−	−	−	−	1
Hemangioma	−	+	+	+	−	+	−	+	+	−	−	−	−	−	6
Nodular hyperplasia	−	+	−	−	−	−	−	−	−	+	−	−	−	−	2
**Ascites**	−	+	+	+	−	−	−	+	−	+	+	−	−	+	7

+, the sign is present; −, the sign is not present; HA—hepatic artery; HV—hepatic vein; IHBD—intrahepatic biliary ducts; PV—portal vein.

## Data Availability

Not applicable.
